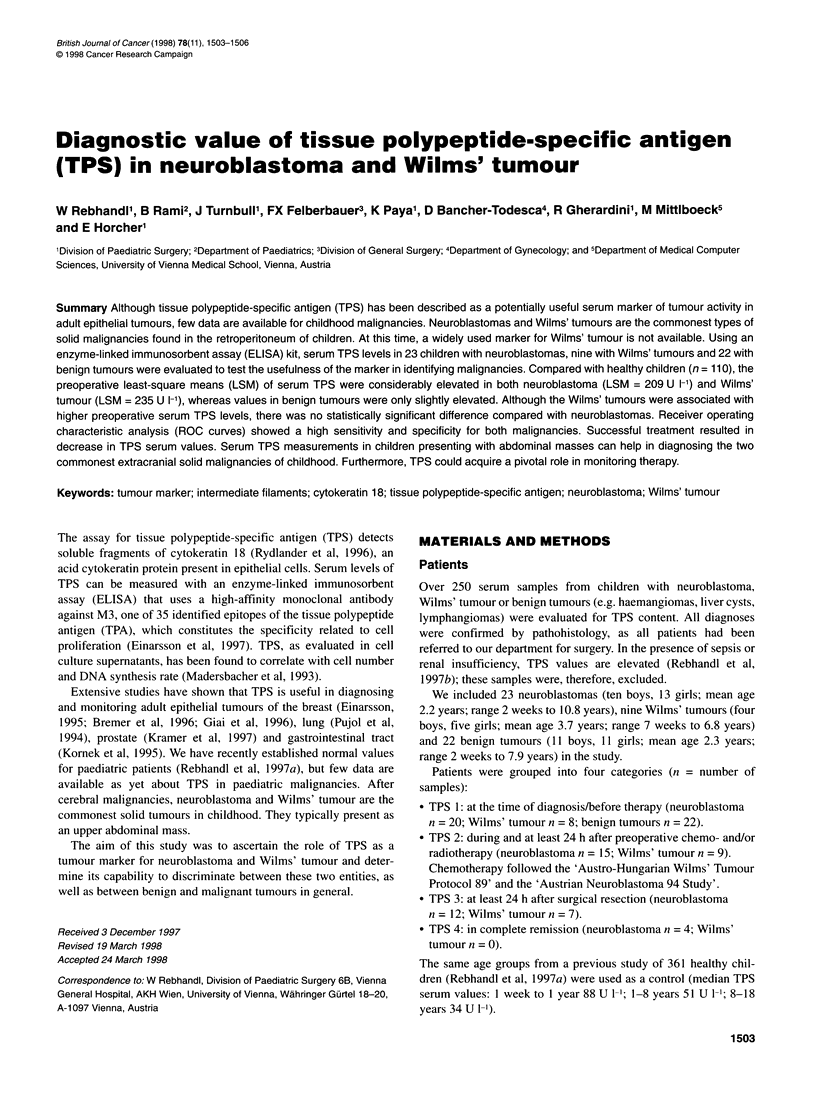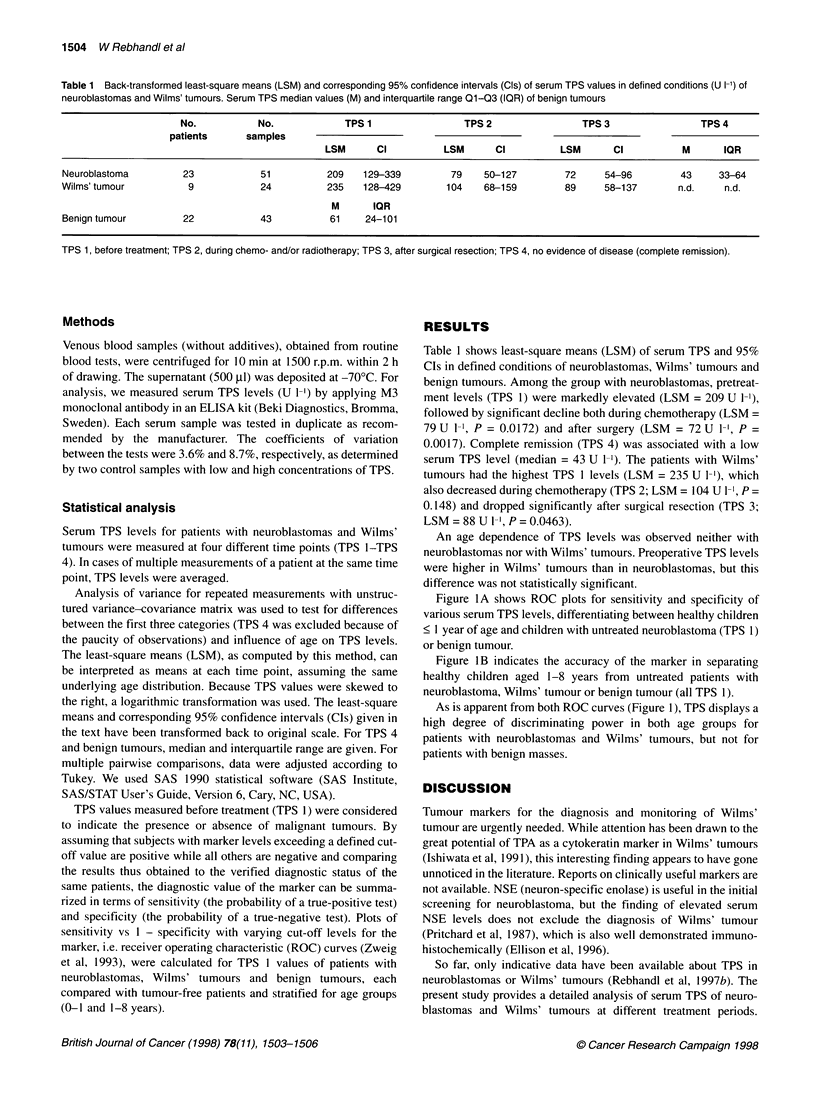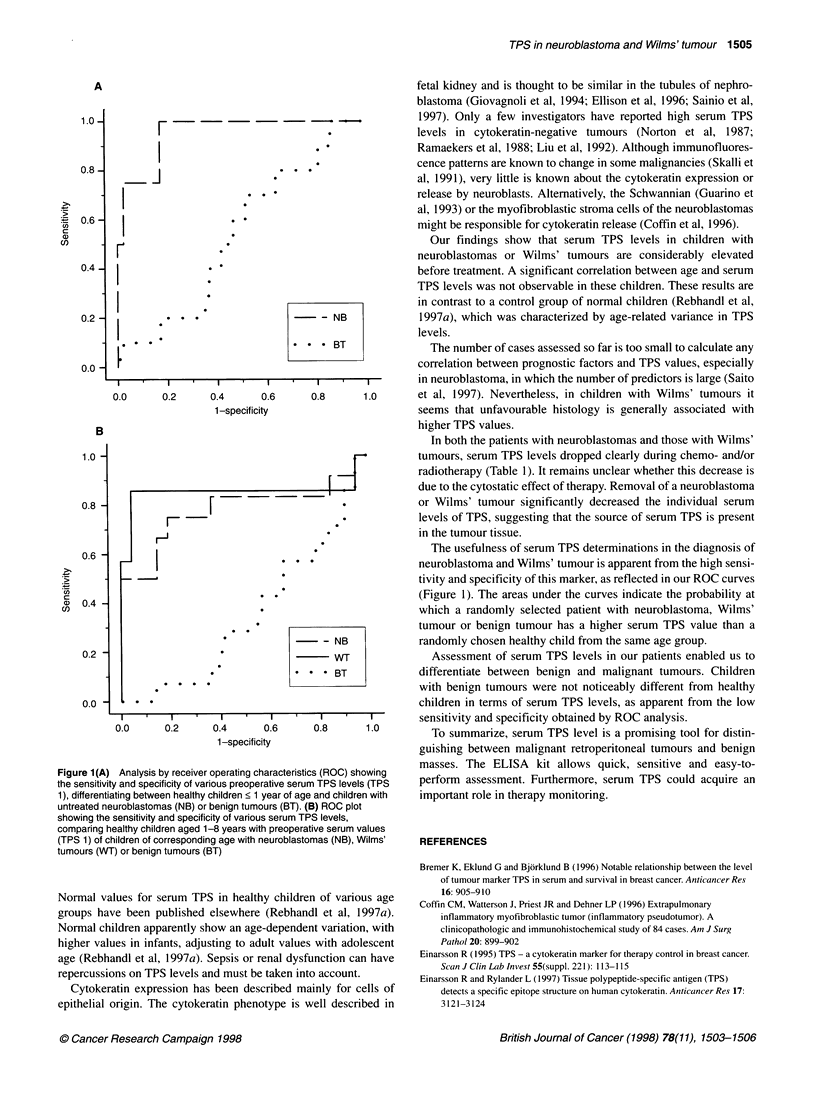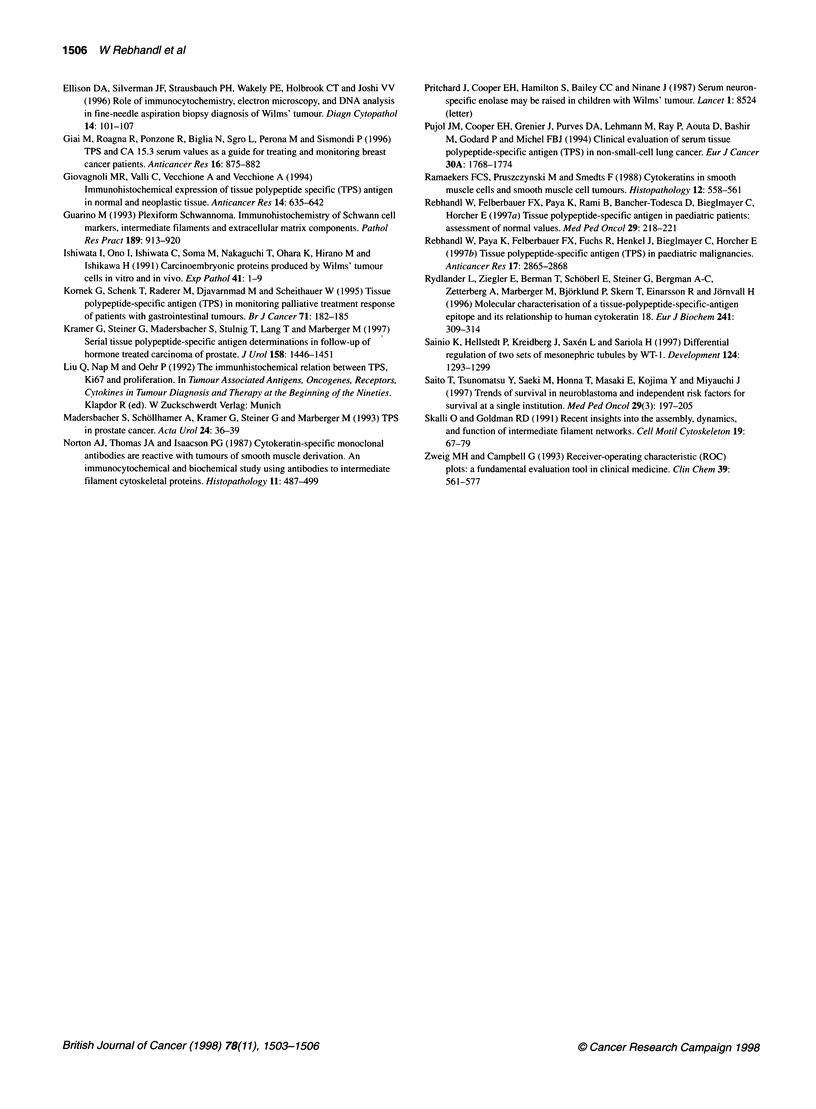# Diagnostic value of tissue polypeptide-specific antigen (TPS) in neuroblastoma and Wilms' tumour.

**DOI:** 10.1038/bjc.1998.713

**Published:** 1998-12

**Authors:** W. Rebhandl, B. Rami, J. Turnbull, F. X. Felberbauer, K. Paya, D. Bancher-Todesca, R. Gherardini, M. Mittlboeck, E. Horcher

**Affiliations:** Division of Paediatric Surgery, University of Vienna Medical School, Austria.

## Abstract

Although tissue polypeptide-specific antigen (TPS) has been described as a potentially useful serum marker of tumour activity in adult epithelial tumours, few data are available for childhood malignancies. Neuroblastomas and Wilms' tumours are the commonest types of solid malignancies found in the retroperitoneum of children. At this time, a widely used marker for Wilms' tumour is not available. Using an enzyme-linked immunosorbent assay (ELISA) kit, serum TPS levels in 23 children with neuroblastomas, nine with Wilms' tumours and 22 with benign tumours were evaluated to test the usefulness of the marker in identifying malignancies. Compared with healthy children (n = 110), the preoperative least-square means (LSM) of serum TPS were considerably elevated in both neuroblastoma (LSM = 209 U l(-1)) and Wilms' tumour (LSM = 235 U l(-1)), whereas values in benign tumours were only slightly elevated. Although the Wilms' tumours were associated with higher preoperative serum TPS levels, there was no statistically significant difference compared with neuroblastomas. Receiver operating characteristic analysis (ROC curves) showed a high sensitivity and specificity for both malignancies. Successful treatment resulted in decrease in TPS serum values. Serum TPS measurements in children presenting with abdominal masses can help in diagnosing the two commonest extracranial solid malignancies of childhood. Furthermore, TPS could acquire a pivotal role in monitoring therapy.


British Journal of Cancer (1998) 78(11), 1503–1506
© 1998 Cancer Research Campaign

### Diagnostic value of tissue polypeptide-specific antigen (TPS) in neuroblastoma and Wilms' tumour

**W Rebhandl[1], B Rami[2], J Turnbull[1], FX Felberbauer[3], K Paya[1], D Bancher-Todesca[4], R Gherardini[1], M Mittlboeck[5] and E Horcher[1]**

[1]Division of Paediatric Surgery; [2]Department of Paediatrics; [3]Division of General Surgery; [4]Department of Gynecology; and [5]Department of Medical Computer Sciences, University of Vienna Medical School, Vienna, Austria

**Summary** Although tissue polypeptide-specific antigen (TPS) has been described as a potentially useful serum marker of tumour activity in adult epithelial tumours, few data are available for childhood malignancies. Neuroblastomas and Wilms' tumours are the commonest types of solid malignancies found in the retroperitoneum of children. At this time, a widely used marker for Wilms' tumour is not available. Using an enzyme-linked immunosorbent assay (ELISA) kit, serum TPS levels in 23 children with neuroblastomas, nine with Wilms' tumours and 22 with benign tumours were evaluated to test the usefulness of the marker in identifying malignancies. Compared with healthy children ($n$ = 110), the preoperative least-square means (LSM) of serum TPS were considerably elevated in both neuroblastoma (LSM = 209 U $l^{-1}$) and Wilms' tumour (LSM = 235 U $l^{-1}$), whereas values in benign tumours were only slightly elevated. Although the Wilms' tumours were associated with higher preoperative serum TPS levels, there was no statistically significant difference compared with neuroblastomas. Receiver operating characteristic analysis (ROC curves) showed a high sensitivity and specificity for both malignancies. Successful treatment resulted in decrease in TPS serum values. Serum TPS measurements in children presenting with abdominal masses can help in diagnosing the two commonest extracranial solid malignancies of childhood. Furthermore, TPS could acquire a pivotal role in monitoring therapy.

**Keywords:** tumour marker; intermediate filaments; cytokeratin 18; tissue polypeptide-specific antigen; neuroblastoma; Wilms' tumour

The assay for tissue polypeptide-specific antigen (TPS) detects soluble fragments of cytokeratin 18 (Rydlander et al, 1996), an acid cytokeratin protein present in epithelial cells. Serum levels of TPS can be measured with an enzyme-linked immunosorbent assay (ELISA) that uses a high-affinity monoclonal antibody against M3, one of 35 identified epitopes of the tissue polypeptide antigen (TPA), which constitutes the specificity related to cell proliferation (Einarsson et al, 1997). TPS, as evaluated in cell culture supernatants, has been found to correlate with cell number and DNA synthesis rate (Madersbacher et al, 1993).

Extensive studies have shown that TPS is useful in diagnosing and monitoring adult epithelial tumours of the breast (Einarsson, 1995; Bremer et al, 1996; Giai et al, 1996), lung (Pujol et al, 1994), prostate (Kramer et al, 1997) and gastrointestinal tract (Kornek et al, 1995). We have recently established normal values for paediatric patients (Rebhandl et al, 1997*a*), but few data are available as yet about TPS in paediatric malignancies. After cerebral malignancies, neuroblastoma and Wilms' tumour are the commonest solid tumours in childhood. They typically present as an upper abdominal mass.

The aim of this study was to ascertain the role of TPS as a tumour marker for neuroblastoma and Wilms' tumour and determine its capability to discriminate between these two entities, as well as between benign and malignant tumours in general.

*Received 3 December 1997*
*Revised 19 March 1998*
*Accepted 24 March 1998*

*Correspondence to:* W Rebhandl, Division of Paediatric Surgery 6B, Vienna General Hospital, AKH Wien, University of Vienna, Währinger Gürtel 18–20, A-1097 Vienna, Austria

### MATERIALS AND METHODS

### Patients

Over 250 serum samples from children with neuroblastoma, Wilms' tumour or benign tumours (e.g. haemangiomas, liver cysts, lymphangiomas) were evaluated for TPS content. All diagnoses were confirmed by pathohistology, as all patients had been referred to our department for surgery. In the presence of sepsis or renal insufficiency, TPS values are elevated (Rebhandl et al, 1997*b*); these samples were, therefore, excluded.

We included 23 neuroblastomas (ten boys, 13 girls; mean age 2.2 years; range 2 weeks to 10.8 years), nine Wilms' tumours (four boys, five girls; mean age 3.7 years; range 7 weeks to 6.8 years) and 22 benign tumours (11 boys, 11 girls; mean age 2.3 years; range 2 weeks to 7.9 years) in the study.

Patients were grouped into four categories ($n$ = number of samples):

- TPS 1: at the time of diagnosis/before therapy (neuroblastoma $n$ = 20; Wilms' tumour $n$ = 8; benign tumours $n$ = 22).
- TPS 2: during and at least 24 h after preoperative chemo- and/or radiotherapy (neuroblastoma $n$ = 15; Wilms' tumour $n$ = 9). Chemotherapy followed the 'Austro-Hungarian Wilms' Tumour Protocol 89' and the 'Austrian Neuroblastoma 94 Study'.
- TPS 3: at least 24 h after surgical resection (neuroblastoma $n$ = 12; Wilms' tumour $n$ = 7).
- TPS 4: in complete remission (neuroblastoma $n$ = 4; Wilms' tumour $n$ = 0).

The same age groups from a previous study of 361 healthy children (Rebhandl et al, 1997*a*) were used as a control (median TPS serum values: 1 week to 1 year 88 U $l^{-1}$; 1–8 years 51 U $l^{-1}$; 8–18 years 34 U $l^{-1}$).

**Table 1** Back-transformed least-square means (LSM) and corresponding 95% confidence intervals (CIs) of serum TPS values in defined conditions (U $l^{-1}$) of neuroblastomas and Wilms' tumours. Serum TPS median values (M) and interquartile range Q1–Q3 (IQR) of benign tumours

| | No. patients | No. samples | TPS 1 | | TPS 2 | | TPS 3 | | TPS 4 | |
|---|---|---|---|---|---|---|---|---|---|---|
| | | | LSM | CI | LSM | CI | LSM | CI | M | IQR |
| Neuroblastoma | 23 | 51 | 209 | 129–339 | 79 | 50–127 | 72 | 54–96 | 43 | 33–64 |
| Wilms' tumour | 9 | 24 | 235 | 128–429 | 104 | 68–159 | 89 | 58–137 | n.d. | n.d. |
| | | | M | IQR | | | | | | |
| Benign tumour | 22 | 43 | 61 | 24–101 | | | | | | |

TPS 1, before treatment; TPS 2, during chemo- and/or radiotherapy; TPS 3, after surgical resection; TPS 4, no evidence of disease (complete remission).

### Methods

Venous blood samples (without additives), obtained from routine blood tests, were centrifuged for 10 min at 1500 r.p.m. within 2 h of drawing. The supernatant (500 µl) was deposited at –70°C. For analysis, we measured serum TPS levels (U $l^{-1}$) by applying M3 monoclonal antibody in an ELISA kit (Beki Diagnostics, Bromma, Sweden). Each serum sample was tested in duplicate as recommended by the manufacturer. The coefficients of variation between the tests were 3.6% and 8.7%, respectively, as determined by two control samples with low and high concentrations of TPS.

### Statistical analysis

Serum TPS levels for patients with neuroblastomas and Wilms' tumours were measured at four different time points (TPS 1–TPS 4). In cases of multiple measurements of a patient at the same time point, TPS levels were averaged.

Analysis of variance for repeated measurements with unstructured variance–covariance matrix was used to test for differences between the first three categories (TPS 4 was excluded because of the paucity of observations) and influence of age on TPS levels. The least-square means (LSM), as computed by this method, can be interpreted as means at each time point, assuming the same underlying age distribution. Because TPS values were skewed to the right, a logarithmic transformation was used. The least-square means and corresponding 95% confidence intervals (CIs) given in the text have been transformed back to original scale. For TPS 4 and benign tumours, median and interquartile range are given. For multiple pairwise comparisons, data were adjusted according to Tukey. We used SAS 1990 statistical software (SAS Institute, SAS/STAT User's Guide, Version 6, Cary, NC, USA).

TPS values measured before treatment (TPS 1) were considered to indicate the presence or absence of malignant tumours. By assuming that subjects with marker levels exceeding a defined cut-off value are positive while all others are negative and comparing the results thus obtained to the verified diagnostic status of the same patients, the diagnostic value of the marker can be summarized in terms of sensitivity (the probability of a true-positive test) and specificity (the probability of a true-negative test). Plots of sensitivity vs 1 – specificity with varying cut-off levels for the marker, i.e. receiver operating characteristic (ROC) curves (Zweig et al, 1993), were calculated for TPS 1 values of patients with neuroblastomas, Wilms' tumours and benign tumours, each compared with tumour-free patients and stratified for age groups (0–1 and 1–8 years).

### RESULTS

Table 1 shows least-square means (LSM) of serum TPS and 95% CIs in defined conditions of neuroblastomas, Wilms' tumours and benign tumours. Among the group with neuroblastomas, pretreatment levels (TPS 1) were markedly elevated (LSM = 209 U $l^{-1}$), followed by significant decline both during chemotherapy (LSM = 79 U $l^{-1}$, $P$ = 0.0172) and after surgery (LSM = 72 U $l^{-1}$, $P$ = 0.0017). Complete remission (TPS 4) was associated with a low serum TPS level (median = 43 U $l^{-1}$). The patients with Wilms' tumours had the highest TPS 1 levels (LSM = 235 U $l^{-1}$), which also decreased during chemotherapy (TPS 2; LSM = 104 U $l^{-1}$, $P$ = 0.148) and dropped significantly after surgical resection (TPS 3; LSM = 88 U $l^{-1}$, $P$ = 0.0463).

An age dependence of TPS levels was observed neither with neuroblastomas nor with Wilms' tumours. Preoperative TPS levels were higher in Wilms' tumours than in neuroblastomas, but this difference was not statistically significant.

Figure 1A shows ROC plots for sensitivity and specificity of various serum TPS levels, differentiating between healthy children ≤ 1 year of age and children with untreated neuroblastoma (TPS 1) or benign tumour.

Figure 1B indicates the accuracy of the marker in separating healthy children aged 1–8 years from untreated patients with neuroblastoma, Wilms' tumour or benign tumour (all TPS 1).

As is apparent from both ROC curves (Figure 1), TPS displays a high degree of discriminating power in both age groups for patients with neuroblastomas and Wilms' tumours, but not for patients with benign masses.

### DISCUSSION

Tumour markers for the diagnosis and monitoring of Wilms' tumour are urgently needed. While attention has been drawn to the great potential of TPA as a cytokeratin marker in Wilms' tumours (Ishiwata et al, 1991), this interesting finding appears to have gone unnoticed in the literature. Reports on clinically useful markers are not available. NSE (neuron-specific enolase) is useful in the initial screening for neuroblastoma, but the finding of elevated serum NSE levels does not exclude the diagnosis of Wilms' tumour (Pritchard et al, 1987), which is also well demonstrated immunohistochemically (Ellison et al, 1996).

So far, only indicative data have been available about TPS in neuroblastomas or Wilms' tumours (Rebhandl et al, 1997*b*). The present study provides a detailed analysis of serum TPS of neuroblastomas and Wilms' tumours at different treatment periods.

*© Cancer Research Campaign 1998*

**Figure 1(A)** Analysis by receiver operating characteristics (ROC) showing the sensitivity and specificity of various preoperative serum TPS levels (TPS 1), differentiating between healthy children ≤ 1 year of age and children with untreated neuroblastomas (NB) or benign tumours (BT). **(B)** ROC plot showing the sensitivity and specificity of various serum TPS levels, comparing healthy children aged 1–8 years with preoperative serum values (TPS 1) of children of corresponding age with neuroblastomas (NB), Wilms' tumours (WT) or benign tumours (BT)

Normal values for serum TPS in healthy children of various age groups have been published elsewhere (Rebhandl et al, 1997*a*). Normal children apparently show an age-dependent variation, with higher values in infants, adjusting to adult values with adolescent age (Rebhandl et al, 1997*a*). Sepsis or renal dysfunction can have repercussions on TPS levels and must be taken into account.

Cytokeratin expression has been described mainly for cells of epithelial origin. The cytokeratin phenotype is well described in fetal kidney and is thought to be similar in the tubules of nephroblastoma (Giovagnoli et al, 1994; Ellison et al, 1996; Sainio et al, 1997). Only a few investigators have reported high serum TPS levels in cytokeratin-negative tumours (Norton et al, 1987; Ramaekers et al, 1988; Liu et al, 1992). Although immunofluorescence patterns are known to change in some malignancies (Skalli et al, 1991), very little is known about the cytokeratin expression or release by neuroblasts. Alternatively, the Schwannian (Guarino et al, 1993) or the myofibroblastic stroma cells of the neuroblastomas might be responsible for cytokeratin release (Coffin et al, 1996).

Our findings show that serum TPS levels in children with neuroblastomas or Wilms' tumours are considerably elevated before treatment. A significant correlation between age and serum TPS levels was not observable in these children. These results are in contrast to a control group of normal children (Rebhandl et al, 1997*a*), which was characterized by age-related variance in TPS levels.

The number of cases assessed so far is too small to calculate any correlation between prognostic factors and TPS values, especially in neuroblastoma, in which the number of predictors is large (Saito et al, 1997). Nevertheless, in children with Wilms' tumours it seems that unfavourable histology is generally associated with higher TPS values.

In both the patients with neuroblastomas and those with Wilms' tumours, serum TPS levels dropped clearly during chemo- and/or radiotherapy (Table 1). It remains unclear whether this decrease is due to the cytostatic effect of therapy. Removal of a neuroblastoma or Wilms' tumour significantly decreased the individual serum levels of TPS, suggesting that the source of serum TPS is present in the tumour tissue.

The usefulness of serum TPS determinations in the diagnosis of neuroblastoma and Wilms' tumour is apparent from the high sensitivity and specificity of this marker, as reflected in our ROC curves (Figure 1). The areas under the curves indicate the probability at which a randomly selected patient with neuroblastoma, Wilms' tumour or benign tumour has a higher serum TPS value than a randomly chosen healthy child from the same age group.

Assessment of serum TPS levels in our patients enabled us to differentiate between benign and malignant tumours. Children with benign tumours were not noticeably different from healthy children in terms of serum TPS levels, as apparent from the low sensitivity and specificity obtained by ROC analysis.

To summarize, serum TPS level is a promising tool for distinguishing between malignant retroperitoneal tumours and benign masses. The ELISA kit allows quick, sensitive and easy-to-perform assessment. Furthermore, serum TPS could acquire an important role in therapy monitoring.

### REFERENCES

Bremer K, Eklund G and Björklund B (1996) Notable relationship between the level of tumour marker TPS in serum and survival in breast cancer. *Anticancer Res* **16**: 905–910

Coffin CM, Watterson J, Priest JR and Dehner LP (1996) Extrapulmonary inflammatory myofibroblastic tumor (inflammatory pseudotumor). A clinicopathologic and immunohistochemical study of 84 cases. *Am J Surg Pathol* **20**: 899–902

Einarsson R (1995) TPS – a cytokeratin marker for therapy control in breast cancer. *Scan J Clin Lab Invest* **55**(suppl. 221): 113–115

Einarsson R and Rylander L (1997) Tissue polypeptide-specific antigen (TPS) detects a specific epitope structure on human cytokeratin. *Anticancer Res* **17**: 3121–3124

Ellison DA, Silverman JF, Strausbauch PH, Wakely PE, Holbrook CT and Joshi VV (1996) Role of immunocytochemistry, electron microscopy, and DNA analysis in fine-needle aspiration biopsy diagnosis of Wilms' tumour. *Diagn Cytopathol* **14**: 101–107

Giai M, Roagna R, Ponzone R, Biglia N, Sgro L, Perona M and Sismondi P (1996) TPS and CA 15.3 serum values as a guide for treating and monitoring breast cancer patients. *Anticancer Res* **16**: 875–882

Giovagnoli MR, Valli C, Vecchione A and Vecchione A (1994) Immunohistochemical expression of tissue polypeptide specific (TPS) antigen in normal and neoplastic tissue. *Anticancer Res* **14**: 635–642

Guarino M (1993) Plexiform Schwannoma. Immunohistochemistry of Schwann cell markers, intermediate filaments and extracellular matrix components. *Pathol Res Pract* **189**: 913–920

Ishiwata I, Ono I, Ishiwata C, Soma M, Nakaguchi T, Ohara K, Hirano M and Ishikawa H (1991) Carcinoembryonic proteins produced by Wilms' tumour cells in vitro and in vivo. *Exp Pathol* **41**: 1–9

Kornek G, Schenk T, Raderer M, Djavarnmad M and Scheithauer W (1995) Tissue polypeptide-specific antigen (TPS) in monitoring palliative treatment response of patients with gastrointestinal tumours. *Br J Cancer* **71**: 182–185

Kramer G, Steiner G, Madersbacher S, Stulnig T, Lang T and Marberger M (1997) Serial tissue polypeptide-specific antigen determinations in follow-up of hormone treated carcinoma of prostate. *J Urol* **158**: 1446–1451

Liu Q, Nap M and Oehr P (1992) The immunhistochemical relation between TPS, Ki67 and proliferation. In *Tumour Associated Antigens, Oncogenes, Receptors, Cytokines in Tumour Diagnosis and Therapy at the Beginning of the Nineties*. Klapdor R (ed). W Zuckschwerdt Verlag: Munich

Madersbacher S, Schöllhamer A, Kramer G, Steiner G and Marberger M (1993) TPS in prostate cancer. *Acta Urol* **24**: 36–39

Norton AJ, Thomas JA and Isaacson PG (1987) Cytokeratin-specific monoclonal antibodies are reactive with tumours of smooth muscle derivation. An immunocytochemical and biochemical study using antibodies to intermediate filament cytoskeletal proteins. *Histopathology* **11**: 487–499

Pritchard J, Cooper EH, Hamilton S, Bailey CC and Ninane J (1987) Serum neuron-specific enolase may be raised in children with Wilms' tumour. *Lancet* **1**: 8524 (letter)

Pujol JM, Cooper EH, Grenier J, Purves DA, Lehmann M, Ray P, Aouta D, Bashir M, Godard P and Michel FBJ (1994) Clinical evaluation of serum tissue polypeptide-specific antigen (TPS) in non-small-cell lung cancer. *Eur J Cancer* **30A**: 1768–1774

Ramaekers FCS, Pruszczynski M and Smedts F (1988) Cytokeratins in smooth muscle cells and smooth muscle cell tumours. *Histopathology* **12**: 558–561

Rebhandl W, Felberbauer FX, Paya K, Rami B, Bancher-Todesca D, Bieglmayer C, Horcher E (1997*a*) Tissue polypeptide-specific antigen in paediatric patients: assessment of normal values. *Med Ped Oncol* **29**: 218–221

Rebhandl W, Paya K, Felberbauer FX, Fuchs R, Henkel J, Bieglmayer C, Horcher E (1997*b*) Tissue polypeptide-specific antigen (TPS) in paediatric malignancies. *Anticancer Res* **17**: 2865–2868

Rydlander L, Ziegler E, Berman T, Schöberl E, Steiner G, Bergman A-C, Zetterberg A, Marberger M, Björklund P, Skern T, Einarsson R and Jörnvall H (1996) Molecular characterisation of a tissue-polypeptide-specific-antigen epitope and its relationship to human cytokeratin 18. *Eur J Biochem* **241**: 309–314

Sainio K, Hellstedt P, Kreidberg J, Saxén L and Sariola H (1997) Differential regulation of two sets of mesonephric tubules by WT-1. *Development* **124**: 1293–1299

Saito T, Tsunomatsu Y, Saeki M, Honna T, Masaki E, Kojima Y and Miyauchi J (1997) Trends of survival in neuroblastoma and independent risk factors for survival at a single institution. *Med Ped Oncol* **29**(3): 197–205

Skalli O and Goldman RD (1991) Recent insights into the assembly, dynamics, and function of intermediate filament networks. *Cell Motil Cytoskeleton* **19**: 67–79

Zweig MH and Campbell G (1993) Receiver-operating characteristic (ROC) plots: a fundamental evaluation tool in clinical medicine. *Clin Chem* **39**: 561–577

© Cancer Research Campaign 1998